# DLK2 Acts as a Potential Prognostic Biomarker for Clear Cell Renal Cell Carcinoma Based on Bioinformatics Analysis

**DOI:** 10.3390/genes13040629

**Published:** 2022-04-01

**Authors:** Man-Gang Lee, Yung-Kuo Lee, Shih-Chung Huang, Chen-Lin Chang, Chou-Yuan Ko, Wen-Chin Lee, Tung-Yuan Chen, Shiow-Jyu Tzou, Cheng-Yi Huang, Ming-Hong Tai, Yu-Wei Lin, Mei-Lang Kung, Ming-Chao Tsai, Yung-Lung Chen, Yi-Chen Chang, Zhi-Hong Wen, Chao-Cheng Huang, Tian-Huei Chu

**Affiliations:** 1 Department of Surgery, Division of Urology, Kaohsiung Armed Forces General Hospital, Kaohsiung 80284, Taiwan; mg2253@yahoo.com.tw; 2 Department of Surgery, Division of Urology, Zuoying Branch of Kaohsiung Armed Forces General Hospital, Kaohsiung 81342, Taiwan; 3 Medical Laboratory, Medical Education and Research Center, Kaohsiung Armed Forces General Hospital, Kaohsiung 80284, Taiwan; u9782001@cmu.edu.tw; 4 Department of Internal Medicine, Division of Cardiology, Kaohsiung Armed Forces General Hospital, Kaohsiung 80284, Taiwan; sghung@gmail.com; 5 Department of Internal Medicine, Division of Cardiology, Tri-Service General Hospital, National Defense Medical Center, Taipei 11490, Taiwan; 6 Institute of Medical Science and Technology, National Sun Yat-sen University, Kaohsiung 80424, Taiwan; changchenling@gmail.com (C.-L.C.); gastroenterokjy@gmail.com (C.-Y.K.); jyu0120@gmail.com (S.-J.T.); 7 Department of Psychiatry, Kaohsiung Armed Forces General Hospital, Kaohsiung 80284, Taiwan; 8 Department of Internal Medicine, Division of Gastroenterology and Hepatology, Kaohsiung Armed Forces General Hospital, Kaohsiung 80284, Taiwan; 9 Department of Internal Medicine, Division of Gastroenterology and Hepatology, Tri-Service General Hospital, National Defense Medical Center, Taipei 11490, Taiwan; 10 Department of Internal Medicine, Division of Nephrology, Kaohsiung Chang Gung Memorial Hospital and Chang Gung University College of Medicine, Kaohsiung 83301, Taiwan; leewenchin@gmail.com; 11 Department of Surgery, Division of Colorectal Surgery, Kaohsiung Armed Forces General Hospital, Kaohsiung 80284, Taiwan; sur045@yahoo.com.tw; 12 Department of Nursing, Kaohsiung Armed Forces General Hospital, Kaohsiung 80284, Taiwan; 13 Institute of Biomedical Sciences, National Sun Yat-sen University, Kaohsiung 80424, Taiwan; zyhuang600@gmail.com (C.-Y.H.); minghongtai@gmail.com (M.-H.T.); 14 Department of Pathology, Kaohsiung Armed Forces General Hospital, Kaohsiung 80284, Taiwan; 15 Center for Neuroscience, National Sun Yat-sen University, Kaohsiung 80424, Taiwan; 16 Department of Radiation Oncology, Kaohsiung Veterans General Hospital, Kaohsiung 813414, Taiwan; marklin1108@gmail.com; 17 Department of Medical Education and Research, Kaohsiung Veterans General Hospital, Kaohsiung 813414, Taiwan; kungmeilang@gmail.com; 18 Department of Internal Medicine, Division of Hepato-Gastroenterology, Kaohsiung Chang Gung Memorial Hospital, Chang Gung University College of Medicine, Kaohsiung 83301, Taiwan; tony0779@gmail.com; 19 Section of Cardiology, Department of Internal Medicine, Kaohsiung Chang Gung Memorial Hospital, Kaohsiung 83301, Taiwan; lung@cgmh.org.tw; 20 Doctoral Degree Program in Marine Biotechnology, National Sun Yat-sen University and Academia Sinica, Kaohsiung 80424, Taiwan; shuikau@gmail.com; 21 Department of Marine Biotechnology and Resources, Asia-Pacific Ocean Research Center, National Sun Yat-sen University, Kaohsiung 80424, Taiwan; wzh@mail.nsysu.edu.tw; 22 Department of Pathology, Kaohsiung Chang Gung Memorial Hospital and Chang Gung University College of Medicine, Kaohsiung 83301, Taiwan; 23 Biobank and Tissue Bank, Kaohsiung Chang Gung Memorial Hospital, Kaohsiung 83301, Taiwan

**Keywords:** clear cell renal cell carcinoma, delta-like 2 homologue, prognosis, biomarker

## Abstract

Clear cell renal cell carcinoma (ccRCC) is the most common RCC subtype with a high mortality. It has been reported that delta-like 1 homologue (DLK1) participates in the tumor microenvironmental remodeling of ccRCC, but the relationship between delta-like 2 homologue (DLK2, a DLK1 homologue) and ccRCC is still unclear. Thus, this study aims to investigate the role of DLK2 in the biological function and disease prognosis of ccRCC using bioinformatics analysis. The TNMplot database showed that DLK2 was upregulated in ccRCC tissues. From the UALCAN analysis, the overexpression of DLK2 was associated with advanced stage and high grade in ccRCC. Moreover, the Kaplan-Meier plotter (KM Plotter) database showed that DLK2 upregulation was associated with poor survival outcome in ccRCC. By the LinkedOmics analysis, DLK2 signaling may participated in the modulation of ccRCC extracellular matrix (ECM), cell metabolism, ribosome biogenesis, TGF-β signaling and Notch pathway. Besides, Tumor Immune Estimation Resource (TIMER) analysis showed that the macrophage and CD8^+^ T cell infiltrations were associated with good prognosis in ccRCC patients. Finally, DLK2 overexpression was associated with the reduced macrophage recruitments and the M1–M2 polarization of macrophage in ccRCC tissues. Together, DLK2 may acts as a novel biomarker, even therapeutic target in ccRCC. However, this study lacks experimental validation, and further studies are required to support this viewpoint.

## 1. Introduction

As one of the most common cancers, renal cell carcinoma (RCC) accounts for around 2–3% of all malignancies [1]. Clear cell renal cell carcinoma (ccRCC) is the most prevalent subtype of kidney cancers, accounting for ~85% of all renal cell carcinomas [2]. Metastases are observed in ~25–30% of ccRCC patients uon first diagnosis; moreover, 20–30% of patients with localized ccRCC have metastases after standard therapy [3]. Both inherited and sporadic ccRCCs are usually associated with structural changes in the chromosome p3 [4], and other potential risk factors include age, gender, lifestyle, complications, drugs, and environmental contaminants [5]. Furthermore, the development of ccRCC is related to multiple gene dysregulations, such as polybromo-1 (PBRM1), BRCA1-associated protein 1 (BAP1), SET domain-containing 2 (SETD2), transcription elongation factor B (SIII), polypeptide 1 (15kDa, elongin C) (TCEB1), lysine (K)-specific demethylase 5C (KDM5C), and Von Hippel–Lindau Tumor Suppressor (VHL) [6]. Furthermore, inactivating the VHL mutation is considered a prevalent risk factor to ccRCC [7], and the dysfunction of VHL promotes neovascularization, which mediated the activation of the HIF/VEGF axis [8]. Although our knowledge of the ccRCC biology has updated, nephrectomy is still the primary option for ccRCC control [9]. For ccRCC patients with late stage or tumor recurrence, some targeted-therapy agents as the first-line drugs, including sorafenib, sunitinib, and aldesleukin, have been used [10]. However, due to different sensitivities to drugs and the genetic background between patients, the survival outcome in ccRCC patients remains poor [11]. Therefore, more potential diagnostic and prognostic biomarkers need to be identified for ccRCC patients in order to guide personalized medicine.

Although Fuhrman nuclear grading and TNM systems are useful prognostic parameters [1], they are still not perfect. Recently, it has been reported that ECM- [12,13], metabolism- [14,15,16], ribosome- [17], and immune-related genes [18,19,20,21] can serve as novel prognostic biomarkers for RCC. Moreover, the immune cell infiltration including T cells and macrophage also impacts disease prognosis in ccRCC [22,23,24,25,26]. These studied indicate that the genetic change in the tumor microenvironment can impact the survival outcome and disease prognosis in ccRCC patients. However, the further validations by more clinical and basic studies were necessary to demonstrate the accuracy of above observations. In recent years, high-throughput genomic analyses, including RNA sequencing and microarray chips, have provided us with big data sets. Computational and bioinformatics techniques have been well applied in the research of various cancers, and have been confirmed to be reliable and powerful for identifying novel biomarkers for cancer diagnosis/prognosis and personalized medicine [27,28,29].

DLK1 (Delta-like 2 Homologue 1) and DLK2 (Delta-like 2 Homologue 2) belong to the EGF-like repeat-containing protein family. Transmembrane DLK1 can also be released to the circulating system after cleavage by tumor necrosis factor α converting enzyme (TACE), but DLK2 lacks the TACE protease cleavage site [30]. DLK1 as an oncoprotein is usually upregulated in many common malignancies (liver, breast, brain, pancreas, colon, and lung). More recently, it has been reported that the overexpression of DLK1 is shown in endocrine-related cancers such as ovarian and adrenocortical carcinoma [31]. Reportedly, DLK1 and DLK2 are two homologous transmembrane proteins, with six extracellular EGF-like repeats that bind with the NOTCH1 receptor and function as endogenous NOTCH inhibitors [32,33,34]. Interestingly, some studies also indicate that DLK1 participates in the tumor progression of neuroblastoma, ovarian high-grade serous carcinoma, and lung cancer mediated the activation of NOTCH1 [35,36,37]. In the DLK2 biology, DLK2 can promote the oncogenic processes of melanoma cells through the inhibition of NOTCH signaling [38]. In kidney disease, DLK2 is upregulated in the injured kidneys after unilateral ureteral obstruction [39], but its biological role in renal inflammation remains unclear. In the RCC study, the DLK1 vaccine in murine models results in the inhibition of RCC growth, but also in the compensatory expression of DLK2 by tumor-associated pericytes [40]. Vaccines targeting both DLK1 and DLK2 show superior antitumor benefits by promoting CD8^+^ T cells infiltrations and tumor vascular normalization. Together, DLK2 may act as a therapeutic target for RCC control. However, the role of DLK2 expression in the prognosis of ccRCC patients remains unclear. To better study the impact on the cancer genetic network of clinical outcomes, genome-wide gene expression databases, such as The Cancer Genome Atlas (TCGA), have been set up to explore and discover large cohorts around the world [41]. TNMplot is a database for the comparison of the gene expression among normal, tumor, and metastatic tissues [42], and as a survival biomarker for multiple cancer types that can be discovered and validated using the Kaplan–Meier plotter (KMplotter) [43]. For the comprehensive analysis of cancer OMICS data, UALCAN and LinkedOmics are widely used for cancer research [44,45]. Systematical analysis of immunocytes recruitments across multiple cancer types can be analyzed by Tumor Immune Estimation Resource (TIMER) [46,47]. Based on bioinformatics analyses by the above-mentioned databases, DLK2 was identified as a potential prognostic biomarker for ccRCC.

## 2. Materials and Methods

### 2.1. Pan-Cancer Analysis

The expression range for the DLK2 gene across all tissues in all available normal and tumor RNA-Seq data was investigated using the Tumor Immune Estimation Resource (TIMER) analysis (https://cistrome.shinyapps.io/timer/) (accessed on 11 December 2021) [46,47].

### 2.2. TNMplot Analysis

The DLK2 expression between paired non-tumor and tumor tissues of ccRCC patients was compared using TNMplot tool (https://tnmplot.com/analysis/) (accessed between 15 August 2021 and 11 December 2021). Moreover, the renal DLK2 levels in the non-ccRCC donor and ccRCC patients were also investigated using TNMplot analysis [42].

### 2.3. UALCAN Analysis

The DLK2 levels in ccRCC tissues with different stages, grades, metastatic status, tumor sub-type, ages, patient races, and genders were analyzed with the UALCAN tool (http://ualcan.path.uab.edu) (accessed on 11 December 2021) [44].

### 2.4. Kaplan–Meier Plotter (KM Plotter) Analysis

The effect of the DLK2 expression on the overall survival and disease recurrence of ccRCC patients was studied using the KMplotter database (https://kmplot.com/analysis/) (accessed on 11 December 2021) [43].

### 2.5. LinkedOmics Database Analysis

The LinkedOmics database (http://www.linkedomics.org/admin.php) (accessed on 11 December 2021) is a web-based platform for analyzing 32 TCGA cancer-associated multi-dimensional datasets [45]. The DLK2-related genes were analyzed statistically using Pearson’s correlation coefficient, presenting in volcano plots, heat maps, or scatter plots. The effect of DLK2 expression on Gene Ontology (GO), including cellular component and molecular function, Kyoto Encyclopedia of Genes and Genomes (KEGG) pathways, Panther pathway, miRNA targets, and transcription factor target in ccRCC were also analyzed with the gene set enrichment analysis (GSEA) using the LinkedOmics database (accessed on 11 December 2021).

### 2.6. TIMER Database Analysis

TIMER is a comprehensive database for systematically analyzing immune cell recruitments across different tumor types from TCGA (https://cistrome.shinyapps.io/timer/) (accessed on 11 December 2021), which includes 10,897 samples across 32 cancer types [46,47]. TIMER uses a deconvolution strategy to deduce the composition of tumor-infiltrating immune cells from the gene expression profiles. The effect of immune cells infiltrations (macrophages, neutrophils, dendritic cells, CD4^+^ T cells, CD8^+^ T cells, and B cells) on the survival outcomes of ccRCC patients was analyzed using the TIMER tool. The correlation of the DLK2 expression and immune cell recruitments in ccRCC was further studied using the TIMER database, and the effect of the DLK2 copy number variation (CNV) on immunocytes recruitments in ccRCC was also investigated. Moreover, the correlation of the DLK2 gene expression and the levels of tumor-associated macrophage (TAMs) markers (M1/M2 markers) was studied in ccRCC patients using the TIMER tool.

### 2.7. Statistical Analysis

Differences between the groups were statistically evaluated using the unpaired Student’s *t* test. The results are showen as mean ± SD, and *p* < 0.05 was considered statistically significant. Moreover, the overall survival outcome and disease recurrence in DLK2^High^ and DLK2^Low^ ccRCC patients was analyzed using the KMplotter tool, and the log-rank test *p* < 0.05 was used to indicate the significance of the survival or recurrence time differences. Pearson’s correlation test was used to analyze the correlation between the DLK2 expression and related gene networks or immune cells infiltrations in ccRCC, and *p* < 0.05 was considered statistically significant

## 3. Results

### 3.1. DLK2 Was Upregulated in the Tumor Tissues of ccRCC Compared with Normal Kidney Tissues

**Figure 1:**
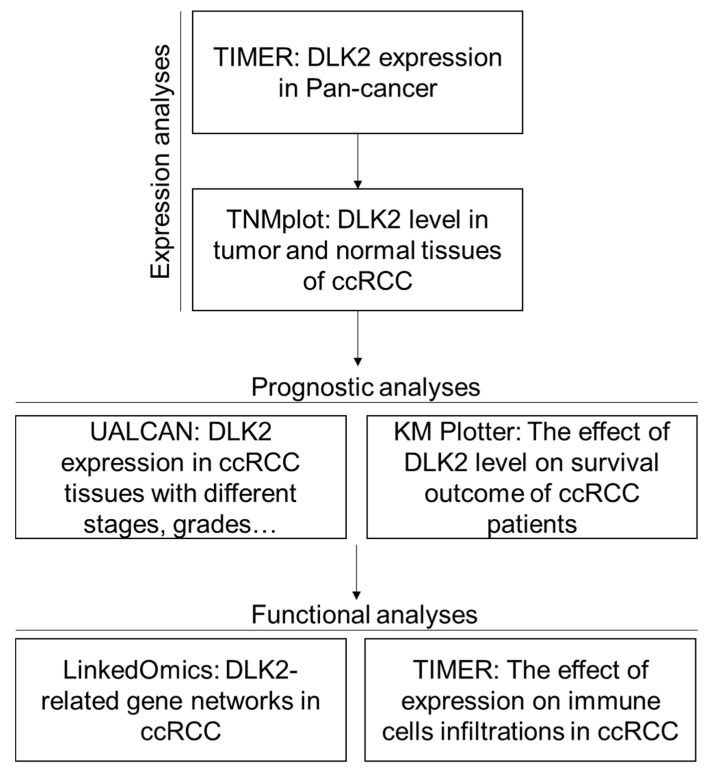
The study workflow indicates the expression, prognostic, and functional analyses that were used to investigate the role of DLK2 in ccRCC.

**Figure 2:**
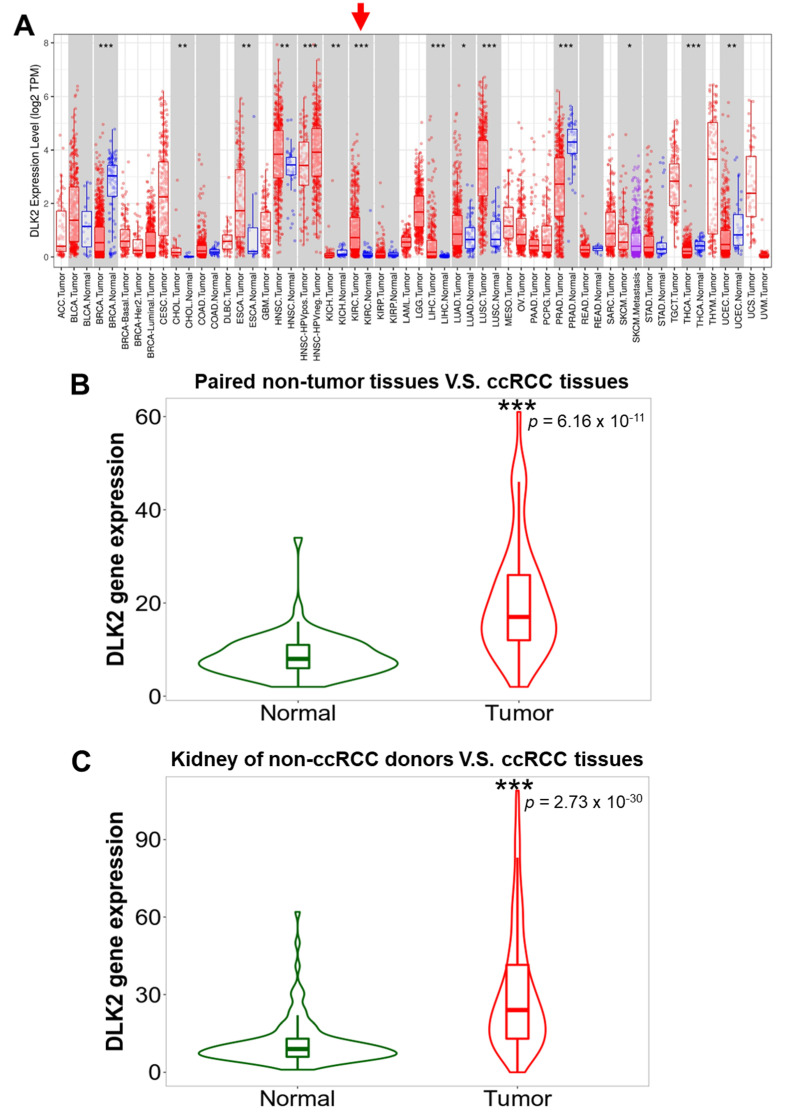
DLK2 is upregulated in ccRCC tissues compared with normal kidney tissues. (**A**) TIMER analysis for DLK2 expression in Pan-cancer. The red arrowhead indicates the ccRCC cohort. (**B**) TNMplot analysis for the DLK2 level in the paired normal and ccRCC tissues. (**C**) TNMplot analysis for DLK2 expression in the renal tissues of non-ccRCC donors and tumor tissues of ccRCC patients. * *p* < 0.05; ** *p* < 0.01; *** *p* < 0.0001.

In this study, we used multiple gene databases to investigate whether DLK2 can serve as a potential prognostic biomarker in ccRCC (Figure 1). Firstly, the DLK2 expression in Pan-cancer was analyzed using the TIMER tool, and TNMplot was used to analyze the DLK2 level in ccRCC tissues and normal renal tissues. For the prognositc analyses, the UALCAN database was used to study the tumoral DLK2 expression profiles in variable ccRCC patients with different disease stages, tumor grades, metastatic status, cancer subtypes, ages, patient races, and genders. The survival outcome and recurrence rate were analyzed in the DLK2^High^ and DLK2^Low^ ccRCC patients using KMplotter. For the molecular functional analyses, the DLK2-related gene networks in ccRCC were identified using the LinkedOmics database, and the effect of the DLK2 level on the immune cell infiltrations of ccRCC was further studied with the TIMER tool. From the Pan-cancer analysis using the TIMER tool, DLK2 was significantly upregulated in the tumor tissues compared with the non-tumor tissues in many cancer types, including ccRCC (Figure 2A) (*** *p* < 0.001). Using the TNMplot analysis, DLK2 was upregulated in paired ccRCC tissues compared with paired non-tumor tissues (Figure 2B) (*** *p* < 0.001), and the expression of DLK2 in the ccRCC tumor was significantly higher than in the kidney from the non-ccRCC donor (Figure 2C) (*** *p* < 0.001). Together, the DLK2 overexpression may participate in the development of ccRCC.

### 3.2. The DLK2 Expression Was Associated with Advanced Tumor Stages/Grades and Worse Overall Survival in ccRCC Patients

**Figure 3:**
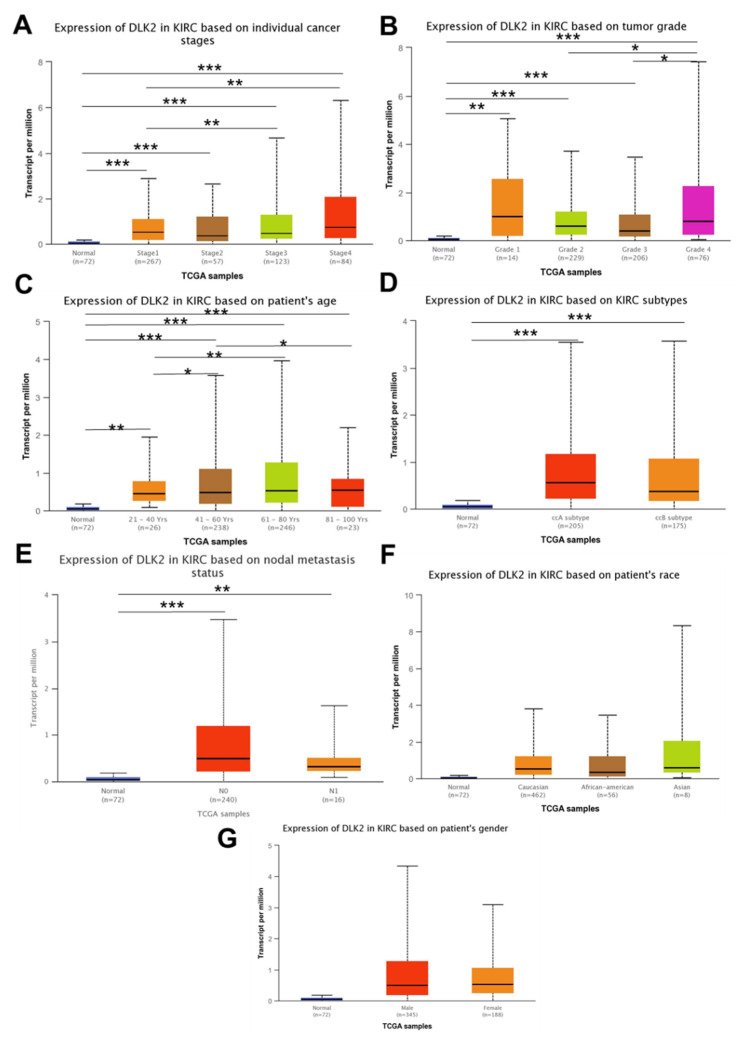
Tumoral DLK2 overexpression is associated with advanced stage and high grade in ccRCC patients. The UALCAN analysis for the tumoral DLK2 level in ccRCC patients with different (**A**) stages, (**B**) grades, (**C**) ages, (**D**) cancer subtypes, (**E**) metastasis status, (**F**) races, and (**G**) genders. * *p* < 0.05; ** *p* < 0.01; *** *p* < 0.0001.

**Figure 4:**
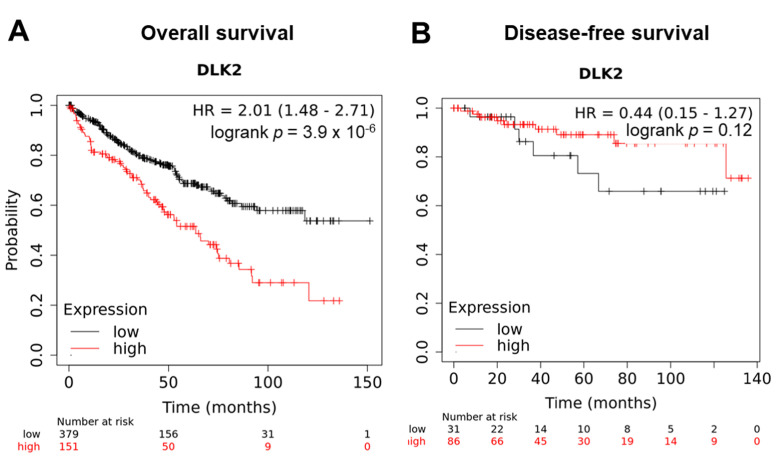
Tumoral DLK2 overexpression is associated with a poor survival outcome in ccRCC patients. The Kaplan–Meier analysis using KMplotter tool for (**A**) overall survival and (**B**) disease-free survival in ccRCC patients with a low or high DLK2 expression.

From the UALCAN analysis, DLK2 was significantly upregulated in the advanced ccRCC stage compared with the early stage (** *p* < 0.01) (Figure 3A). Moreover, DLK2 upregulation was observed in ccRCC tissues with high grades (* *p* < 0.05) (Figure 3B), and an elevated DLK2 level was also shown in the ccRCC tissues of older patients (* *p* < 0.05) (Figure 3C). Furthermore, the cancer subtype, metastatic status, patient race, and gender did not significantly affect the expression of DLK2 in ccRCC tissues (Figure 3D–G). To study the effect of the DLK2 expression on the overall survival and disease-free survival in ccRCC patients, the KMplotter tool was used in this study. From the Kaplan–Meier analysis, ccRCC patients with a higher DLK2 level had a significantly shorter overall survival (*** *p* < 0.001) (Figure 4A), but the DLK2 level in ccRCC tissues did not significantly affect disease-free survival (Figure 4B). Together, DLK2 may serve as a potential prognostic biomarker for ccRCC.

### 3.3. The Gene Clusters Positively and Negatively Correlated with DLK2 Expression Were Identified in ccRCC

**Figure 5:**
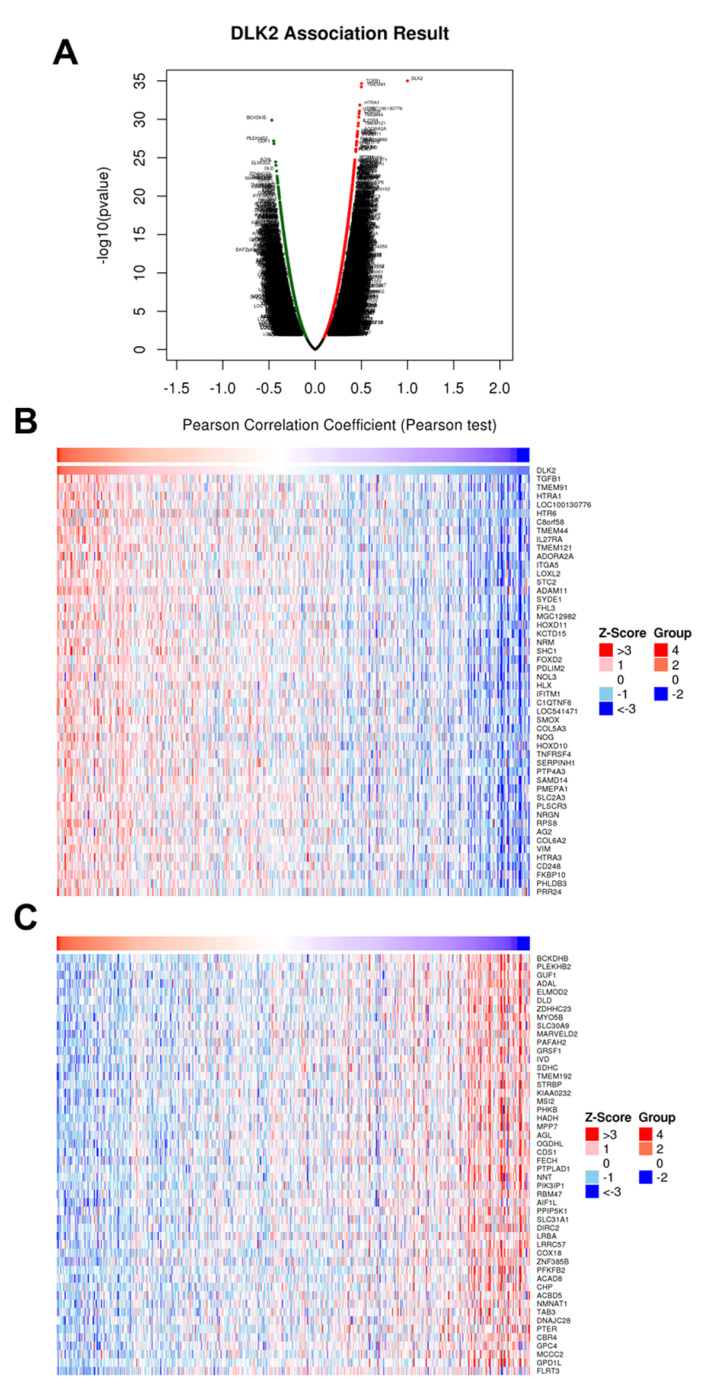
The genes positively and negatively correlated with the DLK2 level in ccRCC are identified using LinkedOmics database. (**A**) The Volcano Plot shows the total genes highly associated with the DLK2 level in ccRCC. Heat maps of the top 50 genes (**B**) positively and (**C**) negatively correlated with DLK2 in ccRCC.

**Figure 6:**
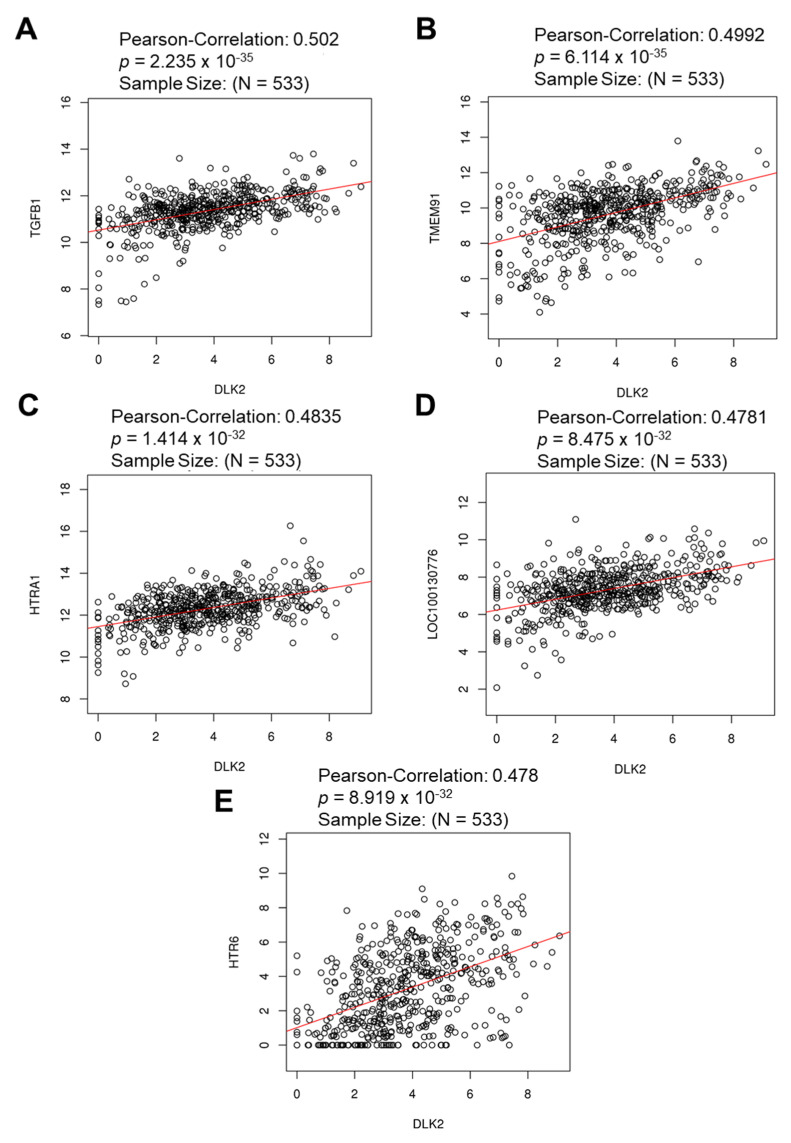
The top five genes positively correlated with the DLK2 level in ccRCC are identified using the LinkedOmics database. Pearson’s correlation analysis for the relationship between DLK2 level and (**A**) TGFβ1, (**B**) TMEM91, (**C**) HTRA1, (**D**) LOC100130776, and (**E**) HTR6.

**Figure 7:**
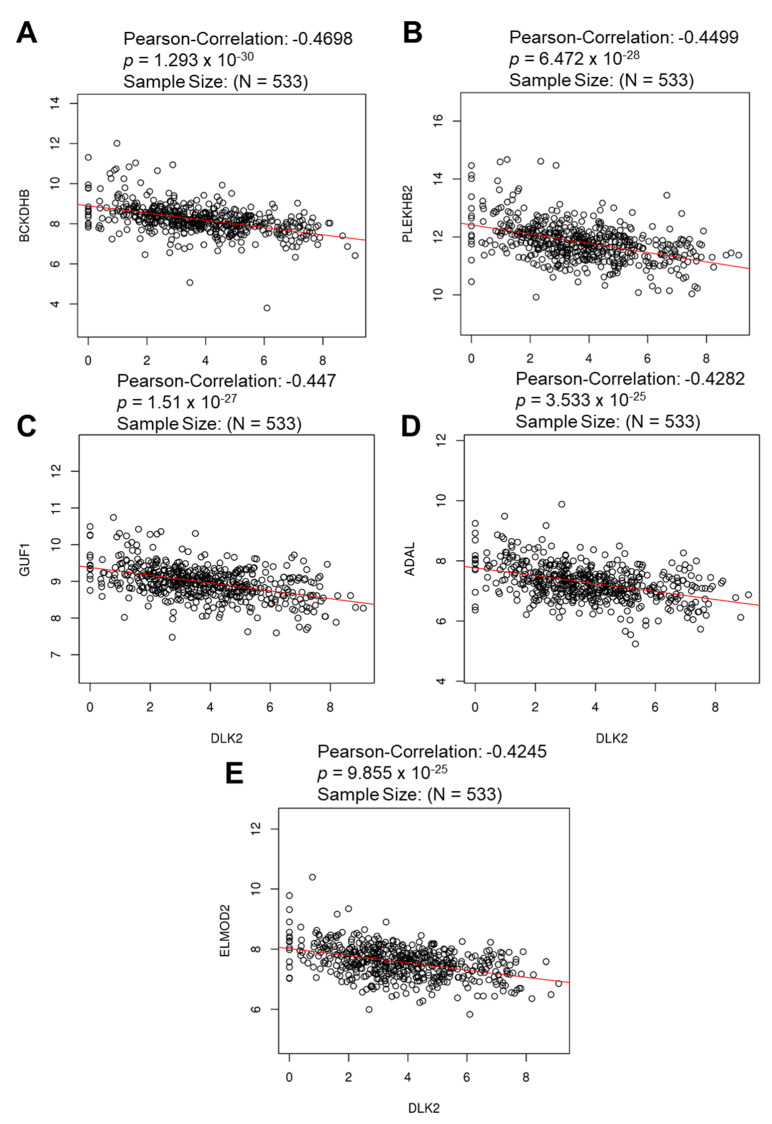
The top five genes negatively correlated with the DLK2 level in ccRCC are identified using LinkedOmics database. Pearson’s correlation analysis for the relationship between DLK2 level and (**A**) BCKDHB, (**B**) PLEKHB2, (**C**) GUF1, (**D**) ADAL, and (**E**) ELMOD2.

From the LinkedOmics database analysis, the Volcano Plot showed the genes highly associated with the DLK2 level in ccRCC (Figure 5A). The top 50 significant genes positively and negatively correlated with the DLK2 level are shown in the heat map in Figure 5B,C. Using Pearson’s correlation analysis, the DLK2 expression showed a strong positive correlation with the transforming growth factor β 1 (TGFβ1) (*r* = 0.502, *p* = 2.235 × 10^−35^), transmembrane protein 91 (TMEM91) (*r* = 0.4992, *p* = 6.114 × 10^−35^), HtrA serine peptidase 1 (HTRA1) (*r* = 0.4992, *p* = 6.114 × 10^−35^), AGAP2 antisense RNA 1 (AGAP2-AS1) (LOC100130776) (*r* = 0.4781, *p* = 8.475 × 10^−32^), and 5-Hydroxytryptamine receptor 6 (HTR6) (*r* = 0.478, *p* = 8.919 × 10^−32^) levels in human ccRCC tissues (Figure 6). In addition, the expressions of branched chain keto acid dehydrogenase E1 subunit β (BCKDHB) (*r* = −0.4698, *p* = 1.293 × 10^−30^), pleckstrin homology domain containing B2 (PLEKHB2) (*r* = −0.4499, *p* = 6.472 × 10^−28^), GTP binding elongation factor GUF1 (GUF1) (*r* = −0.447, *p* = 1.51 × 10^−27^), adenosine deaminase like (ADAL) (*r* = −0.4282, *p* = 3.533 × 10^−25^), and ELMO domain containing 2 (ELMOD2) (*r* = −0.4245, *p* = 9.855 × 10^−25^) were highly and negatively correlated to the DLK2 levels in the ccRCC tumors (Figure 7).

To further identify the molecular targets of DLK2 in ccRCC, we analyzed the potent miRNA and transcription factor targets using the LinkedOmics tool. The most correlated microRNA-targets of DLK2 in ccRCC were GGGGCCC, miR-296 (*p* = 0), CCAGGGG, miR-331 (p = 0), AGCTCCT, miR-28 (*p* = 0.002294), CATGTAA, miR-496 (*p* = 0.032258), and TTTTGAG, miR-373 (*p* = 0.045455) (Table 1). Furthermore, the most correlated transcript factor-targets of DLK2 in ccRCC were V$LFA1_Q6 (genes with 3’UTR containing motif GGGSTCWR, which matches annotation for ITGAL) (*p* = 0), V$MAZR_01 (genes with 3’UTR containing motif NSGGGGGGGGMCN, which matches annotation for ZNF278) (*p* = 0), V$VDR_Q3 (genes with 3’UTR containing motif GGGKNARNRRGGWSA, which matches annotation for VDR) (*p* = 0), V$ZIC3_01 (genes with 3’UTR containing motif NGGGKGGTC, which matches annotation for ZIC3) (*p* = 0), and GGGNNTTTCC_V$NFKB_Q6_01 (genes with 3′UTR containing motif GGGNNTTTCC, which matches annotation for NFκB) (*p* = 0).

### 3.4. DLK2-Associated Functional Enrichment Items in ccRCC Were Identified Using the LinkedOmics Tool

**Figure 8:**
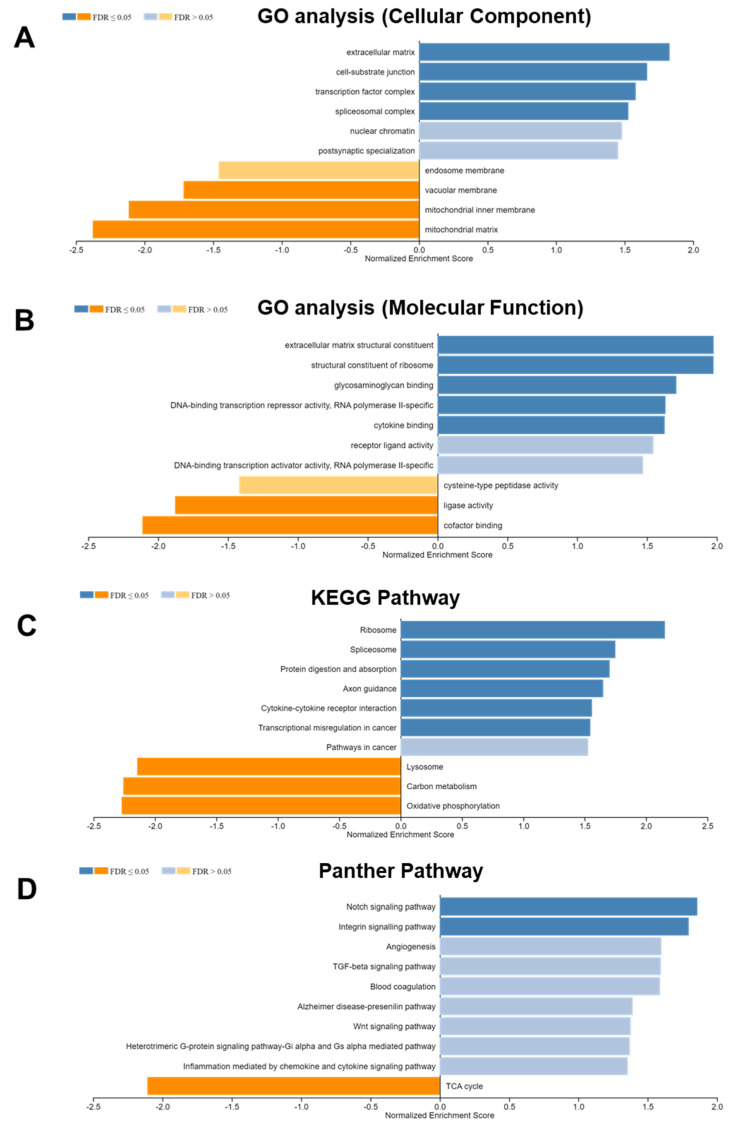
The gene sets positively and negatively correlated with the DLK2 level in ccRCC are identified using the LinkedOmics tool. The (**A**,**B**) GO, (**C**) KEGG, and (**D**) Panther pathways analyses for DLK2 in ccRCC. The blue bars indicate the gene clusters positively correlated with DLK2. The orange bars indicate the gene clusters negatively correlated with DLK2.

In order to examine the DLK2-related functions in ccRCC, we performed an enrichment analysis using the LinkedOmics tool. The Gene Ontology (GO) analysis for cellular components showed that DLK2 was mainly involved in the positive regulation of the extracellular matrix-, cell-substrate junction-, transcription factor complex-, spliceosomal complex-, nuclear chromatin-, and postsynaptic specialization-associated gene clusters (Figure 8A), and endosome membrane-/vacuolar membrane-/mitochondria inner membrane-/mitochondria matrix-related gene expressions were negatively correlated with the DLK2 level in ccRCC. In the GO analysis for molecular function, DLK2 may positively regulate the genes participating in the extracellular matrix structural constituent, structural constituent of ribosome, glycosaminoglycan binding, DNA-binding transcription repressor activity (RNA polymerase II-specific), cytokine binding, receptor ligand activity, and DNA-binding transcription activator activity (RNA polymerase II-specific) (Figure 8B), and the cell molecular functions for cysteine-type peptidase activity/ligase activiy/cofactor binding may be negatively modulated by DLK2 in ccRCC. Moreover, the Kyoto Encyclopedia of Genes and Genomes (KEGG) pathway analysis showed that DLK2 were positively modulated in the gene networks that participated in ribosome biogenesis, splicesome formation, protein digestion/absorption, axon guidance, cytokine–cytokine receptor interaction, transcriptional misregulation of cancer, and pathways in cancer (Figure 8C), and lysosome-/carbon metabolism-/oxidative phosphorylation-related gene networks was negatively regulated by DLK2 in ccRCC. By the Panther pathway analysis, DLK2 may positively modulate the Notch signaling pathway, integrin signaling pathway, angiogenesis, TGF-β signaling pathway, blood coagulation, Alzheimer disease-presenilin pathway, Wnt signaling pathway, heterotrimeric G-protein signaling pathway/Gs α mediated pathway, and inflammation mediated by chemokine/cytokine signaling pathway (Figure 8D), and the tricarboxylic acid (TCA) cycle was negatively modulated by DLK2 in ccRCC. Together, the extracellular matrix (ECM), cell metabolism, ribosome biogenesis, TGF-β signaling, and Notch pathway may have participated in the DLK2-promoted oncogenic processes in ccRCC.

### 3.5. DLK2 Expression Was Negatively Correlated with the Macrophages Infiltrations and Positively Correated with the M1 to M2 Polarization of Macrophages in ccRCC

**Figure 9:**
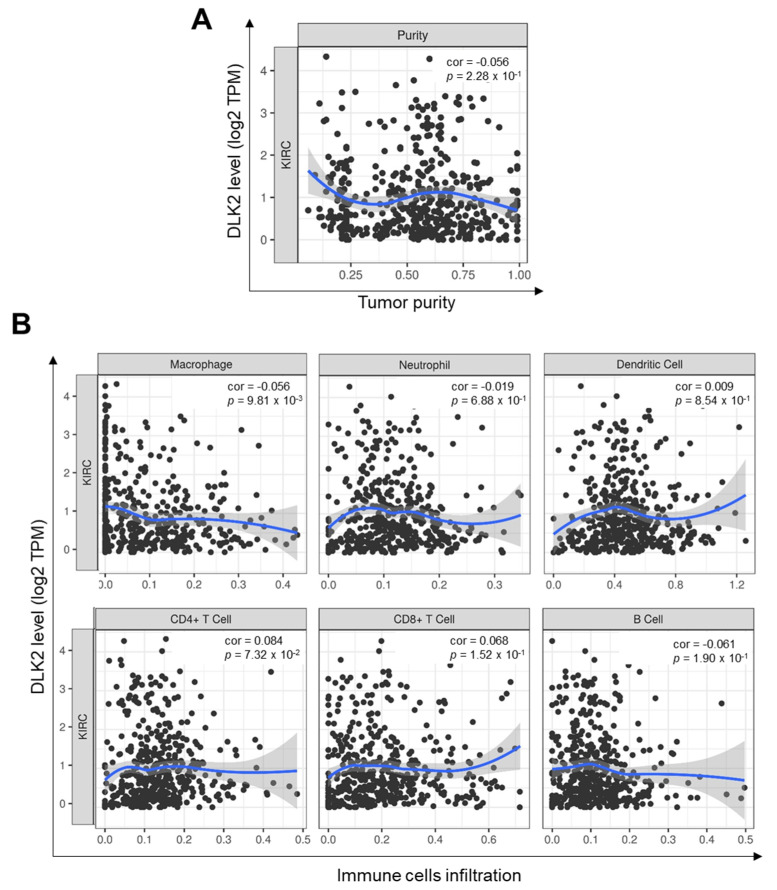
The correlation between DLK2 expression and immune cells infiltrations in ccRCC is analyzed using the TIMER database. (**A**) The correlation between the DLK2 level and tumor purity in ccRCC. (**B**) The correlation between the DLK2 expression and the recruitments of macrophages, neutrophils, dendritic cells, CD4^+^ T cells, CD8^+^ T cells, and B cells in ccRCC tissues.

**Figure 10:**
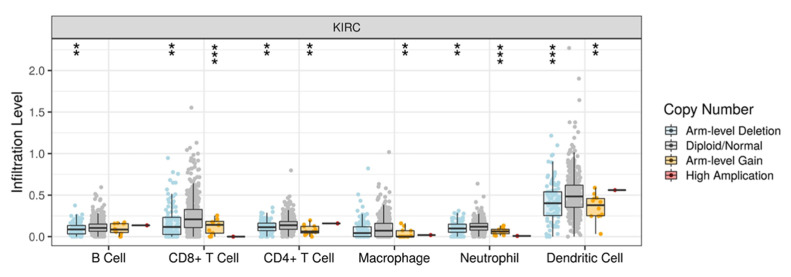
The copy number variation (CNV) of DLK2 affects the immune cells infiltration in ccRCC based on the TIMER analysis. The effect of DLK2 CNV on the B cells, CD8^+^ T cells, CD4^+^ T cells, macrophages, neutrophils, and dendritic cells in ccRCC. ** *p* < 0.01; *** *p* < 0.0001.

Previous studies have reported that immune cells infiltrations impact the disease prognosis in ccRCC patients [22,48]. The TIMER database was used to comprehensively study the effect of tumor immune cells recruitment on the survival outcome in ccRCC patients (Table 2). The Cox proportional hazard model showed that the tumor infiltrations of macrophages and CD8^+^ T cells were significantly associated with a reduced mortality rate in ccRCC patients (* *p* < 0.05). Furthermore, the tumor infiltrations of neutrophils, dendritic cells, CD4^+^ T cells, and B cells did not significantly impact the survival outcome of ccRCC patients. From the Pearson’s correlation test, the DLK2 level was not significantly associated with tumor purity, which indicates that the tumor microenvironment is also a source of DLK2 expression (Figure 9A). Moreover, the DLK2 level was significantly associated with reduced macrophage infiltrations (** *p* < 0.01), and the recruitments of neutrophils, dendritic cells, CD4^+^ T cells, CD8^+^ T cells, and B cells were not significantly correlated with DLK2 expression in ccRCC (Figure 9B). From the SCNA module of the TIMER tool, arm-level deletion of the DLK2 gene caused the reduction of B cell (** *p* < 0.01), CD8^+^ T cells (** *p* < 0.01), CD4^+^ T cells (** *p* < 0.01), neutrophil (** *p* < 0.01), and dendritic cell infiltrations (** *p* < 0.01) in ccRCC (Figure 10). Second, arm-level gain of DLK2 gene reduced CD8^+^ T cells (*** *p* < 0.001), CD4^+^ T cells (** *p* < 0.01), macrophage (** *p* < 0.01), neutrophil (*** *p* < 0.001), and dendritic cell infiltrations (** *p* < 0.01) in tumor tissue. In the macrophage recruitment, the results from the Pearson’s correlation analysis and SCNA module were consistent in this study.

**Figure 11:**
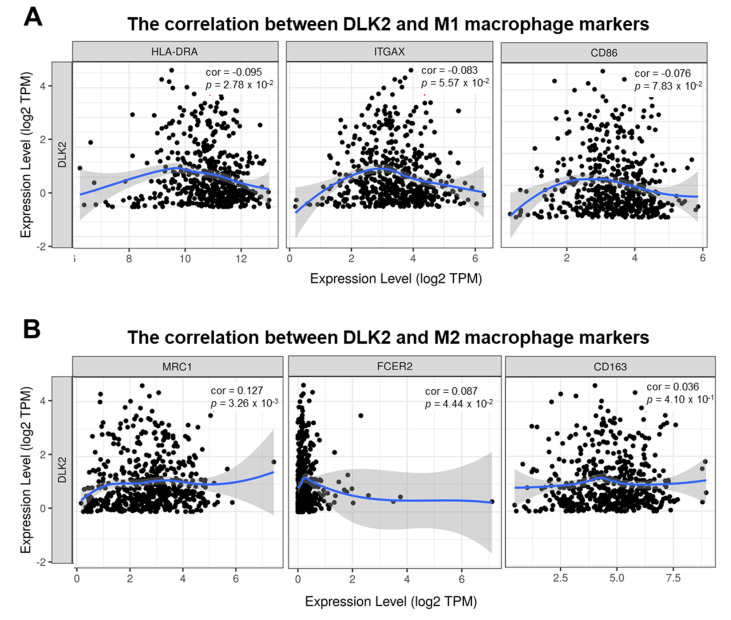
The DLK2 expression is associated with the M1 to M2 polarization of macrophages in ccRCC based on the TIMER analysis. The correlation between DLK2 level and the expressions of (**A**) M1 or (**B**) M2 macrophage markers in ccRCC tissues.

Reportedly, M1 macrophages (anti-tumor phenotype) are associated with a favorable outcome, while M2 macrophages (pro-tumor phenotype) indicate a worse outcome in RCC [22]. Thus, the correlation between the DLK2 level and the expressions of M1/M2 macrophage markers in ccRCC was investigated using the TIMER tool. According to the Pearson’s correlation analysis, the tumor DLK2 level was negatively correlated with the expressions of M1 macrophage markers such as HLA class II histocompatibility antigen, DR α chain (HLA-DRA) (* *p* = 0.0278), CD11c (integrin α X, ITGAX) (*p* = 0.0557), and CD86 (*p* = 0.0783) [49,50] (Figure 11A). Moreover, the expressions of M2 macrophage markers such as CD206 (mannose receptor C-type 1, MRC1) (** *p* = 0.00326) and CD23 (Fc epsilon receptor II, FCER2) (* *p* = 0.0444) were positively associated with the DLK2 level in ccRCC (Figure 11B). Thus, the DLK2 expression may impact not only macrophage infiltrations, but also M1 to M2 polarization in ccRCC.

## 4. Discussion

**Figure 12:**
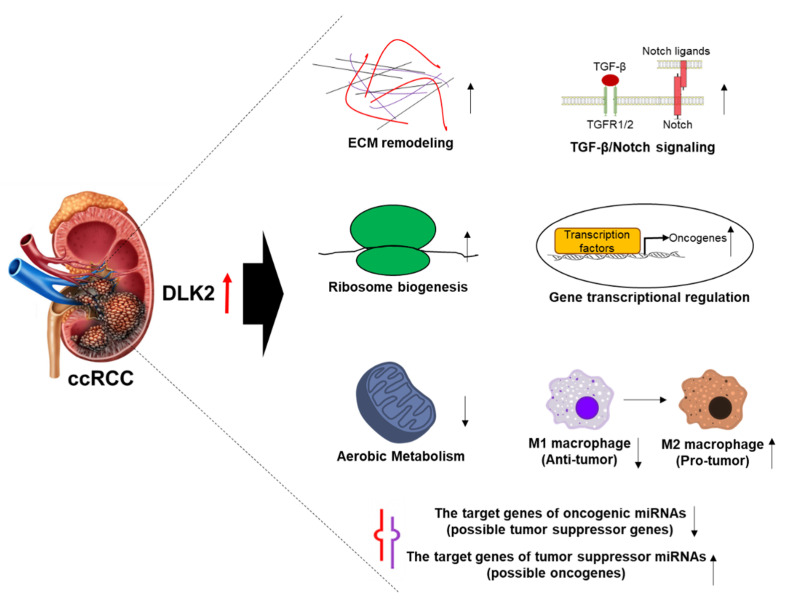
Hypothetical model for the role of DLK2 in ccRCC.

Based on this meta-analysis by multiple gene expression databases, DLK2 was upregulated in ccRCC tumors compared with normal renal tissues. In addition, DLK2 overexpression was associated with an advanced stage and a poorly differentiated grade, and was correlated with a worse survival outcome in ccRCC patients, indicating that DLK2 may play an oncogenic role and serve as a promising and novel prognostic factor for ccRCC. Through the molecular and functional analysis of the bioinformatics, we herewith proposed a mechanistic model for the oncogenic processes of DLK2 in ccRCC (Figure 12). DLK2 may participate in ECM remodeling, ribosome biogenesis, the activation of TGF-β/Notch oncogenic signaling, gene transcriptional regulation, M1 to M2 polarization of macrophage, and the increment of tumor suppressor miRNAs targets (possible oncogenes). Moreover, aerobic metabolism in the mitochondria and the transcription of oncogenic miRNAs targets (possible tumor suppressor genes) may be shut down by DLK2 signaling. Together, DLK2 may act as a potent therapeutic target for ccRCC control by modulating the oncogenic processes of tumor cell and the tumor microenvironment.

From the Panther pathway and Pearson’s correlation analyses based on the LinkedOmics database, DLK2 signaling may positively regulate the TGF-β1 and Notch signaling pathways, and it has been reported that an extensive cross-talk between the TGF-β1 and Notch signaling cascades is associated with the aggressiveness of ccRCC [51]. It has been reported that Notch activation can inhibit the TCA cycle in Drosophila wing discs and human microvascular cells [52]. Importantly, the TGF-β/HDAC7 signaling pathway can repress oxidative phosphorylation in RCC [53], and the administration of TGF-β inhibitor restores the expression of TCA cycle enzymes and inhibits tumor progression in the orthotopic RCC model. In ECM biology, both TGF-β1 and Notch signaling play a critical role in ECM remodeling [54,55], and TGF-β1-promoted ECM remodeling impacts the survival outcome in ccRCC patients [56]. Importantly, it has been reported that DLK1 signaling promotes the upregulation of matrix metalloproteinase-9 (MMP9) through the activation of Notch1 signaling [37]. In ribosome biogenesis, many ribosome-related proteins can serve as prognostic biomarkers and therapeutic targets in RCC [17,57,58], and some studies indicate that TGF-β1 and Notch are involved in the modulation of ribosome-related pathways [59,60]. Thus, TGF-β1 and Notch signaling may participate in DLK2-promoted ribosome biogenesis in ccRCC. Reportedly, M2 macrophage infiltration is a risk factor for poor prognosis in ccRCC patients, and M2 macrophage can serve as a potential biomarker for prognosis and novel targets for immunotherapy in ccRCC [26]. Moreover, both TGF-β1-induced Snail signaling and the Jagged1-mediated Notch pathway can also promote the M2 polarization of the macrophage in the tumor microenvironment [61,62], and this means that DLK2-activated TGF-β1 and Notch signaling may participate in the M2 polarization of the macrophage in the ccRCC tumor microenvironment.

In addition to the TGF-β1 level, the expressions of HTRA1 and AGAP2-AS1 were highly and positively correlated with the DLK2 level in ccRCC tissues. Previous studies indicate that HTRA1 participates in the neovascularization mediated activation of Notch1 signaling [63]. Moreover, AGAP2-AS1 (LOC100130776) can promote the radioresistance of lung cancer cells [64] and act as an independent predictor of poor survival in ccRCC patients [65]. Thus, HTRA1 and AGAP2-AS1 signaling pathways may be involved in the DLK2-promoted oncogenic processes of ccRCC cells. Furthermore, the expressions of GUF1 and ELMOD2 were significantly and negatively correlated with the DLK2 level in ccRCC. It has been reported that GUF1 and ELMOD2 promote mitochondria protein synthesis and fusion, respectively [66,67], and these genes may participate in the DLK2-modulated mitochondria metabolism. Another gene, PLEKHB2 (also called evectin-2), negatively correlated with the DLK2 level, plays a critical role in the YAP oncogenic pathway of proliferating cells [68], and it is also downregulated in colon cancer [69]. In the miRNA analysis, the expressions of miR-296, miR-331, and miR-28 targets (possible oncogenes) were positively correlated with the DLK2 level, and previous studies indicate that miR-296, miR-331, and miR-28 sever as tumor suppressors [70,71,72]. Furthermore, the DLK2 level was negatively correlated with the expressions of miR-496 and miR-373 targets (possible tumor suppressor genes). In addition, it has been reported that miR-496 and miR-373 also play an oncogenic role in cancer progression [73,74]. From the LinkedOmics database analysis for transcription factor targets positively associated with the DLK2 level, the expressions of CD11A (also called LFA1 or ITGAL)-, Zinc finger protein 278 (ZNF278, also called MAZR)-, vitamin D receptor (VDR)-, Zic Family Member 3 (ZIC3)-, and NFκB-related transcription targets were highly associated with the DLK2 level in ccRCC. Moreover, CD11A, ZNF278, VDR, ZIC3, and NFκB also play critical roles in the modulation of oncogenic processes in many type cancers [75,76,77,78,79]. Together, DLK2-modulated miRNA targets and transcription factor targets may play a crucial role in the carcinogenesis of ccRCC. In addition to surgery and radiotherapy, first-line systemic treatments including tyrosine kinase inhibitors (TKIs) and immunotherapy are also used for ccRCC control [80]. However, the therapeutic efficacy of TKIs and immune checkpoint inhibitors for ccRCC remains unsatisfactory due to drug resistance [81]. Thus, DLK2 targeting may serve as a novel therapeutic strategy for ccRCC management. In the future, more clinical, animal, and cell studies are required for further validation of DLK2’s role in ccRCC.

## 5. Conclusions

In conclusion, this systematic review and meta-analysis illustrated that DLK2 may constitute a novel prognostic biomarker in ccRCC based on multiple gene expression databases. The upregulation of DLK2 in tumor tissues was associated with advanced stage, high tumor grade, and poor survival outcome in ccRCC patients. At the same time, we also found that DLK2 may serve as a potent oncogene in ccRCC by regulating ECM remodeling, mitochondria metabolism, ribosome biogenesis, TGF-β signaling, and Notch pathway. However, this study lacks experimental evidence, and the actual effect of DLK2 on the oncogenic processes and disease prognosis of ccRCC should be further investigated using a series of in vitro, in vivo, and clinical studies. If DLK2 is a poor prognostic factor and oncogene in ccRCC, and it may act as a novel biomarker or therapeutic target for ccRCC management in the future.

## Figures and Tables

**Table 1:** The miRNA- and transcription factor-target networks highly associated with DLK2 in ccRCC (LinkedOmics).

Enriched Category	Gene Set	Normalized Enrichment Score	FDR	Leading Edge Number	*p* Value
miRNA Target	GGGGCCC, miR-296	1.865499	0.002889	25	0
	CCAGGGG, miR-331	1.643038	0.038283	29	0
	AGCTCCT, miR-28	1.555229	0.119423	27	0.002294
	CATGTAA, miR-496	−1.258861	0.475076	32	0.032258
	TTTTGAG, miR-373	−1.157875	0.485488	31	0.045455
Transcription Factor Target	V$LFA1_Q6	1.723831	0.003247	73	0
	V$MAZR_01	1.724924	0.003788	60	0
	V$VDR_Q3	1.748140	0.004132	79	0
	V$ZIC3_01	1.727236	0.004546	94	0
	GGGNNTTTCC_V$NFKB_Q6_01	1.751883	0.005510	50	0

**Table 2:** Cox proportional hazard model showing hazard ratios for ccRCC conferred by variables.

Variable	Coefficient	HR	95% CI	*p* Value
Macrophage	−2.774	0.062	0.006–0.647	* 0.020
Neutrophil	3.211	24.809	0.389–1582.755	0.130
Dendritic cell	1.119	3.062	0.517–18.131	0.217
CD4^+^ T cell	−0.524	0.592	0.039–8.902	0.705
CD8^+^ T cell	−1.741	0.175	0.037–0.837	* 0.029
B cell	−0.600	0.549	0.022–13.757	0.714

* *p* < 0.05.

## Data Availability

The data presented in this study are available upon request from the corresponding author. The data sets used in this study can be accessed at https://cistrome.shinyapps.io/timer/ (TIMER), https://tnmplot.com/analysis/ (TNMplot), http://ualcan.path.uab.edu (UALCAN), and https://kmplot.com/analysis/ (KMplotter), http://www.linkedomics.org/admin.php (LinkedOmics). These gene expression databases were accessed on 11 December 2021.
